# Implementing shared decision-making for hospitalized patients with schizophrenia: a qualitative study of nurse and patient perspectives

**DOI:** 10.3389/fpsyt.2026.1873569

**Published:** 2026-08-06

**Authors:** Bin Xu, Yanxia Guo, Yan Han, Xiaofang Lin, Miaomiao Li, Lili Wu, Xueyuan Hou

**Affiliations:** 1 Huai’an Third People’s Hospital, Huaian, China; 2 School of Nursing and Midwifery, Jiangsu College of Nursing, Huaian, China

**Keywords:** nurse-patient relations, person-centered care, psychiatric nursing, qualitative research, schizophrenia, shared decision-making

## Abstract

**Background:**

Shared decision-making (SDM) is fundamental to person-centered psychiatry, yet its implementation in schizophrenia care remains limited and poorly understood. Existing research predominantly examines SDM from either healthcare provider or patient perspectives, overlooking the integrated perspectives from both sides within clinical settings.

**Objective:**

To explore shared decision-making experiences in inpatient schizophrenia care from the integrated perspectives of nurses and patients on the same clinical unit.

**Methods:**

A phenomenological qualitative study was conducted in a tertiary psychiatric hospital in Jiangsu Province, China. Semi-structured interviews were conducted with 12 psychiatric nurses and 12 inpatients with schizophrenia between April and December 2024. Data were analyzed using Colaizzi’s seven-step phenomenological analysis framework.

**Results:**

Both nurses and patients recognized the value of SDM, yet implementation encountered multidimensional barriers. Four interconnected themes emerged (1): Perceptions of SDM and role positioning—nurses recognized the value of SDM and assumed multiple facilitator roles, while patients’ perceived capacity for participation fluctuated with clinical status, requiring dynamic assessment and progressive engagement; (2) Attitudes and trust relationships influencing SDM implementation—nurses displayed diverse attitudes ranging from proactive support to explicit opposition, while trust between clinicians and patients was identified as the cornerstone of effective SDM; (3) Practical challenges in SDM implementation—clinical and ethical complexities, compounded by ineffective information communication, created significant barriers to SDM; and (4) Internal and external support systems affecting SDM—family involvement exerted dual influences of facilitation and paternalistic substitution, while pervasive social stigma further hindered patient participation.

**Conclusions:**

Implementing SDM in schizophrenia care is a systematic endeavor shaped by the interplay of conceptual, relational, communicative, and contextual factors. Advancing patient-centered psychiatry requires a dual transformation: empowering nurses as skilled facilitators while actively activating patients as central participants, supported by trust-based relationships, tailored information tools, and integrated family-social resources.

## 1 Introduction

Schizophrenia is a serious mental disorder that affects a person’s thinking, perception, self-experience, cognition, volition, affect and behavior (1). Schizophrenia is the most common of all psychiatric disorders and is prevalent in all cultures across the world. About 15% of new admission in mental hospitals is schizophrenic patients. It has been estimated that patients diagnosed as having schizophrenia occupy 50% of all mental hospital beds (2). The chronic and debilitating nature of this illness necessitates long-term treatment engagement, making the quality of clinical decision-making processes particularly consequential for patient outcomes (3).

Shared decision-making (SDM) is often defined as a collaborative process in which clinicians and patients work together to select tests, treatments, management or support packages, based on clinical evidence and the patient’s informed preferences (4, 5), SDM represents a paradigm shift from paternalistic models of care toward partnership-based approaches. For individuals with schizophrenia, meaningful involvement in treatment decisions carries significant potential to enhance therapeutic adherence, strengthen working alliances with healthcare providers, and foster recovery-oriented outcomes (6).

Despite these recognized benefits, the implementation of SDM in schizophrenia treatment remains markedly limited and poorly understood. Patients with schizophrenia face unique barriers to participation, including illness-related cognitive impairments, diminished insight, reduced decision-making capacity during acute phases, and internalized stigma (7, 8). Concurrently, healthcare providers may harbor doubts about patients’ competence to participate in decisions, leading to exclusionary practices even when patients possess the capacity for involvement. These compounded challenges render schizophrenia care a particularly complex context for SDM implementation.

Existing research has predominantly examined SDM from singular perspectives—either focusing on healthcare providers’ attitudes and practices or exploring patients’ experiences in isolation (9, 10). While valuable, these unidimensional approaches fail to capture the dynamics and reciprocal influences that shape SDM processes within clinical encounters. Notably, studies integrating the views of both providers and patients from the same clinical context are exceptionally rare, representing a critical gap in the literature.

Nurses occupy a uniquely positioned role in the SDM process. As the clinical staff with the most frequent and sustained contact with hospitalized patients, psychiatric nurses serve as frontline facilitators of patient engagement, coordinators of information exchange, and advocates for patient autonomy (11, 12). Their perceptions, attitudes, and communicative practices substantially influence whether and how SDM unfolds in daily care. Simultaneously, patients’ subjective experiences and perceptions of involvement constitute the ultimate benchmark for evaluating SDM effectiveness. Understanding both perspectives within the same clinical unit is essential for identifying congruence and divergence in how SDM is understood, experienced, and enacted.

To address this gap, the present study innovatively incorporates both nurses and hospitalized patients with schizophrenia as core participants from the same clinical setting. Using phenomenological qualitative methods, we seek to (1): explore nurses’ cognitions, attitudes, and practical experiences regarding SDM implementation; (2) examine patients’ awareness of SDM, their experiences of participation in treatment decisions, and perceived facilitators and barriers; and (3) integrate these dual perspectives to reveal the complex interactions and systemic factors affecting SDM practice in inpatient psychiatric settings. This multi-dimensional analysis aims to provide a comprehensive foundation for developing clinically applicable, contextually responsive SDM models that can enhance person-centered care for individuals living with schizophrenia.

## 2 Methods

### 2.1 Study design

A phenomenological qualitative study design was employed to explore shared decision-making experiences from the perspectives of nurses and patients in inpatient psychiatric settings. Phenomenology is particularly appropriate for this inquiry as it seeks to understand the essence of lived experiences and the meanings individuals attribute to phenomena within their lifeworld (13). Given that SDM involves deeply personal and relational experiences that are shaped by subjective perceptions of trust, capacity, and autonomy, a phenomenological approach enables us to capture the richness and complexity of how both nurses and patients make sense of their involvement in clinical decision-making. This study adopts an inductive approach, consistent with phenomenological inquiry, allowing themes and patterns to emerge from the data rather than being imposed by pre-existing theoretical frameworks (14).

### 2.2 Sampling and recruitment

Participants were recruited via purposive sampling from the same severe mental disorder ward of a tertiary psychiatric hospital in Jiangsu Province during two phases in 2024. Between April and June 2024, clinical nurses were recruited. Inclusion criteria were (1): ≥5 years of psychiatric nursing experience; (2) basic understanding of SDM concepts; (3) good communication skills; and (4) voluntary informed consent. Excluded were those who withdrew during interviews. From October to December 2024, hospitalized patients meeting ICD-10 criteria for schizophrenia were recruited from the same ward. Inclusion criteria were: (1) hospitalization >2 weeks; (2) minimum primary education level and basic communication ability; (3) age ≥18 years; and (4) willingness to participate autonomously. Patients with severe physical comorbidities, substance abuse, or significant cognitive impairment hindering communication were excluded. While nurses and patients were recruited independently and at different time periods (parallel sampling) without one-to-one matching, all participants were drawn from the same clinical unit, providing a shared institutional context for comparing perspectives. The sequential recruitment approach was adopted to minimize disruption to clinical care and to avoid potential cross-contamination between the two participant groups.

### 2.3 Data collection

Semi-structured interview guides were developed separately for nurses and patients based on an extensive literature review and team discussions. The initial drafts of the semi-structured interview guides for both nurses and patients were reviewed by a panel of five experts, including chief psychiatrists, senior psychiatric nurses, and a qualitative research specialist, to ensure content validity and relevance. Subsequently, pilot interviews were conducted with two nurses and two patients (who were not included in the final sample) to assess the clarity, comprehensibility, and logical flow of the questions, leading to minor refinements in wording and sequencing.

The final nurse interview guide explored participants’ understanding of SDM, current practices in clinical settings, perceived influencing factors, personal attitudes toward patient involvement, and suggestions for improving SDM implementation. The patient interview guide examined patients’ awareness of SDM, their experiences and feelings regarding participation in treatment decisions, perceived facilitators and barriers to involvement, and their expectations of healthcare professionals. The semi-structured interview guides for both nurses and patients are shown in **Supplementary Material**.

All interviews were conducted face-to-face in private, quiet rooms within the hospital to ensure confidentiality and minimize distractions. Prior to each interview, written informed consent was obtained from all participants. Participants were provided with a detailed Participant Information Sheet explaining the study purpose, procedures, risks, benefits, confidentiality measures, and their right to withdraw at any time without affecting their care. All participants signed a written consent form. Immediately before each interview commenced, the interviewer verbally re-explained the key study information and confirmed the participant’s ongoing willingness to participate; this verbal confirmation was audio-recorded as part of the interview recording. The nurse interviews were conducted by a head nurse with 20 years of psychiatric nursing experience at the same hospital but did not supervise any participants (she worked on a different clinical unit). To mitigate the potential influence of her senior status, she reassured participants that responses were anonymous, would not affect their employment, and that candid views were encouraged. A non-judgmental, exploratory stance was maintained throughout. The second researcher involved in analysis (who did not conduct the interviews) provided independent critical review to identify potential interpretive bias. Each nurse interview lasted 20–40 minutes, with a total of 12 interviews completed, amounting to 421 minutes of audio recording.

Patient interviews were conducted by an experienced provincial-level psychiatric nurse skilled in communicating with individuals with severe mental illness. The interviewer paid close attention to participants’ non-verbal cues, adjusted communication style according to patients’ conditions, and used probing and clarifying questions to elicit rich, detailed responses. Each interview lasted 30–50 minutes. Each patient participated in one interview, with the exception of one participant who completed a second interview to allow deeper exploration of emergent themes related to family involvement in decision-making. A total of 13 interviews were completed, producing 392 minutes of recordings.

All audio recordings were transcribed verbatim within 24 hours in Chinese. Representative quotations selected for publication were translated into English through a rigorous process. One of the researchers with a bilingual native Chinese speaker with professional English proficiency performed the initial translation. A second bilingual researcher, who was not involved in the initial translation, independently back-translated the English quotations into Chinese. The research team then compared the back-translated Chinese with the original transcripts, resolving any discrepancies or semantic nuances through consensus discussion. This iterative translation and verification process ensured that the final English quotations accurately preserved participants’ original meanings and contextual nuances.

### 2.4 Data analysis

Data were analyzed using Colaizzi’s seven-step phenomenological analysis framework (15, 16). This involved: repeatedly reading transcripts; extracting significant statements; formulating meanings; clustering themes; developing exhaustive descriptions; producing fundamental structures; and returning findings to participants for verification. Data collection and analysis proceeded iteratively. After every three interviews, the research team held a formal review meeting to compare newly generated codes and themes against those already identified. Thematic saturation was considered achieved when three consecutive interviews yielded no new codes or themes, and the team reached consensus that existing themes were sufficiently rich to address the research objectives. A final review of the entire dataset confirmed that no additional themes were identifiable.

For nurse data, two researchers (including the interviewer) analyzed the data together. A total of 40,106 Chinese characters of nurse transcripts were generated for analysis, from which 372 meaningful codes were derived. For patient data, two researchers independently coded the transcripts. The patient transcripts yielded 37,862 characters, from which 289 codes were generated. NVivo 14.0 software was used to assist data management and analysis for both groups. Discrepancies in coding were resolved through research team discussions.

### 2.5 Rigor and trustworthiness

To ensure rigor and trustworthiness, multiple strategies were employed throughout the research process: uniform interviewer training; building rapport with participants; using neutral, open-ended questions; recording non-verbal cues; independent coding and triangulation among researchers; peer debriefing by experts not involved in interviews; and member checking. For member checking, preliminary summaries of interpreted data were returned to a subset of participants for verification. These participants were asked to review whether the researchers’ interpretations accurately reflected their experiences and perspectives. All confirmed the accuracy of the interpretations, with some providing additional contextual details that further enriched the analysis. This process ensured that the findings remained grounded in participants’ lived experiences and reduced the potential for researcher bias.

### 2.6 Ethics approval

The study protocol was approved by the Ethics Committee of Huai’an Third People’s Hospital (IRB No: 2024-02). Written informed consent was obtained from all participants prior to data collection, and verbal re-consent was audio-recorded at the start of each interview to reaffirm participants’ ongoing willingness to participate. In reporting our findings, we adhered to the Consolidated Criteria for Reporting Qualitative Research (COREQ) guidelines.

## 3 Results

A total of 12 nurses (N1–N12) and 12 hospitalized patients with schizophrenia (P1–P12) participated in this study. Among the nurse participants, 3 were male and 9 were female, aged 24 to 48 years (mean = 32.37 ± 5.16). Their nursing experience ranged from 5 to 28 years (median = 10 years). Educational backgrounds included Bachelor’s degrees (n = 11) and college diploma (n = 1). Professional titles were Senior (n = 3), Intermediate (n = 8), and Junior (n = 1).

Among the patient participants, 7 were male and 5 were female, aged 23 to 61 years (mean = 43.58 ± 13.21). All patients met ICD-10 criteria for schizophrenia, with diagnoses including paranoid schizophrenia (n = 7), undifferentiated schizophrenia (n = 3), simple schizophrenia (n = 1), and schizophrenia (n = 1). Educational levels ranged from primary school to Bachelor’s degree. Marital status varied among single (n = 5), married (n = 5), and divorced (n = 2). Length of hospital stay ranged from 16 to 33 days (mean = 23.67 ± 6.21).

**Figure 1:**
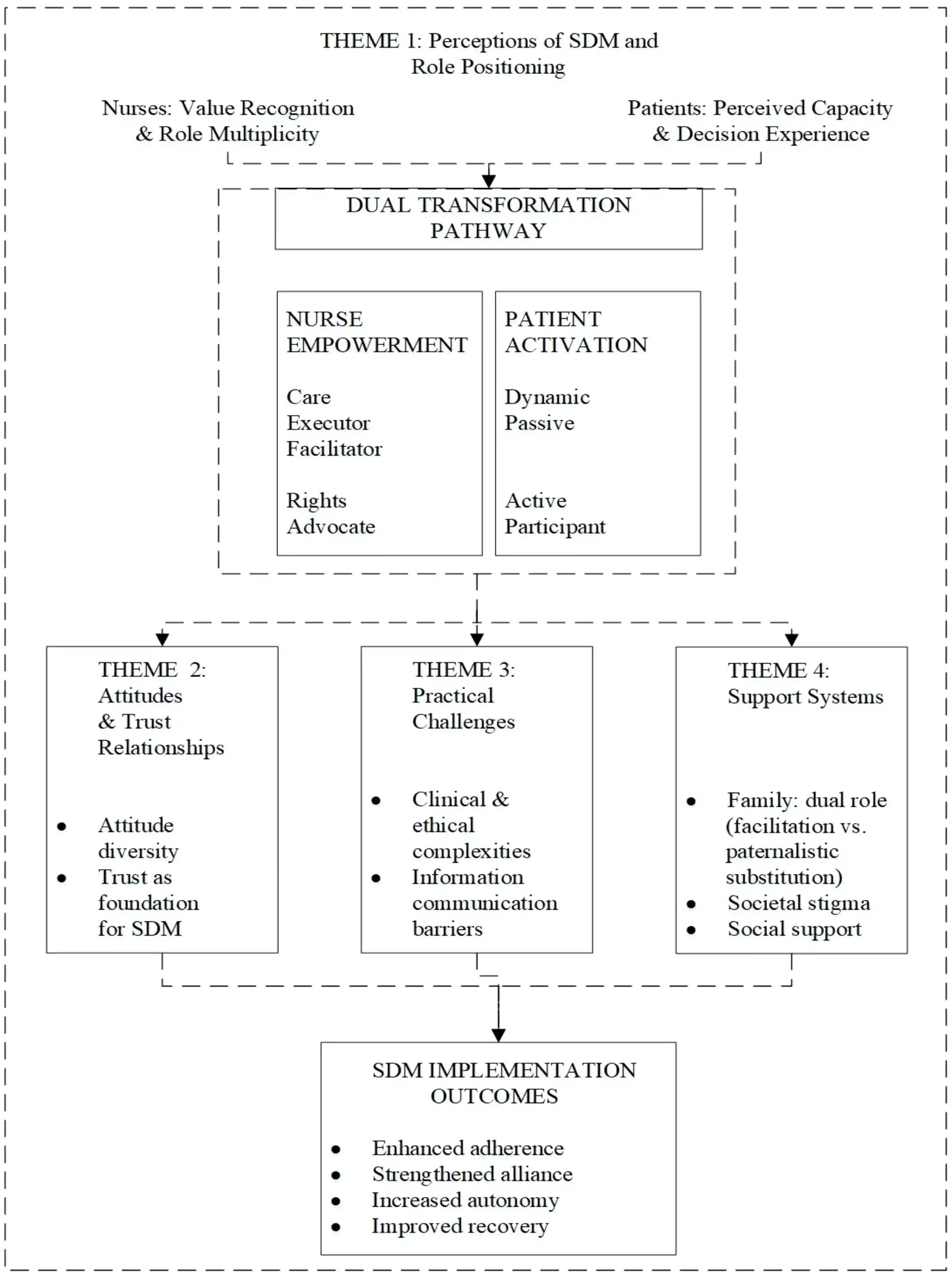
Thematic model and dual transformation pathway of SDM implementation in schizophrenia inpatient care.

Based on Colaizzi’s phenomenological analysis framework, we identified four primary themes: (1) Perceptions of SDM and role positioning, which integrates nurses’ multifaceted understanding of their roles and patients’ first-hand experiences in decision-making, revealing the multidimensional nature of SDM in clinical practice; (2) Attitudes and trust relationships influencing SDM implementation, highlighting the diversity in nursing staff attitudes and the profound impact of nurse-patient trust on SDM effectiveness; (3) Practical challenges in SDM implementation, focusing on key barriers hindering the effective application of SDM in clinical settings; and (4) Internal and external support systems affecting SDM. Influencing factors and thematic structure of SDM in schizophrenia inpatient care in **Table 1**. The Thematic model and dual transformation pathway of SDM implementation in schizophrenia inpatient care was shown in **Figure 1**.

**Table 1:** Influencing factors and thematic structure of SDM in schizophrenia inpatient care.

Theme	Subtheme	Representative quotes
Perceptions of SDM and role positioning	Value recognition and role multiplicity	“Involving patients in decision-making can improve their self-identity and promote more in-depth communication.” “ Implementing shared decision-making is the optimal model for doctor-patient communication. It can empower patients with autonomy, enhance their disease management capabilities, and improve their clinical outcomes.” “This is a patient-centered treatment model that emphasizes the joint participation of doctors, patients, and their families in treatment choices. It is particularly suitable for complex diseases requiring long-term management like schizophrenia.”
	Patients’ perceived capacity and decision-making experience	“I hope to discuss my condition with the doctor together.” “During periods of disease stability, I possess a certain degree of insight and am able to participate in treatment choices … During acute episodes, when insight is impaired, the decision-making authority often shifts to family members or the medical team.” “We gradually participate in discussions, such as adjusting medication dosage, choosing psychotherapy … or formulating rehabilitation plans.”
Attitudes and trust relationships influencing SDM implementation	Diversity in nursing staff attitudes	“Patients’ judgment differs significantly from the norm, making it difficult for them to reach sound decisions.” “We should establish a trust relationship with patients with mental disorders, demonstrating respect, understanding, and care towards them, so that patients can feel safe and comfortable, and thus be more willing to participate in the decision-making process.” “I hope the doctor praises me more. Last time I said, “I want to reduce the medication because I feel very sleepy every day during the daytime.” The doctor didn’t criticize me; instead, he said, “This shows you are responsible for yourself.” I was very happy … I felt respected.”
	Central role of trust relationships	“The doctors and nurses are highly professional; I feel reassured following their guidance.” “If a mutually trusting and good relationship can be established with the patient, then the patient will find it easier and be more willing to accept clinical shared decision-making.” “Shared decision-making in schizophrenia is not “letting patients choose freely,” but finding the intersection of the goals of doctors and patients under professional guidance. The core is to establish trust, using small-step exploration to reduce decision-making pressure, and ultimately enhance treatment confidence and quality of life.”
Practical challenges in SDM implementation	Clinical and ethical contexts	“Medication may lead to either improvement or deterioration of symptoms. When patients’ judgment is impaired, SDM becomes particularly challenging.” “On the patient side, some patients have severe psychiatric symptoms, and prolonged treatment medication generally leads to cognitive dysfunction, resulting in an inability to correctly understand the content when we implement shared decision-making.” “Doctors and patients have different perspectives when evaluating the pros and cons of treatment options. The patient’s choice may not achieve their own best interests and could delay the optimal treatment timing.”
	Barriers to information communication	“The doctor said it might cause ‘tardive dyskinesia,’ but I didn’t understand what that meant,” “In clinical practice, because we are often busy, we neglect to assess the patient’s level of understanding. During this time, patient education may also be merely verbal, with insufficiently diverse formats, leading to low patient acceptance rates.” “When doctors explain using simple analogies (like “medication is like a key; you need to find the right lock”), and avoid being too technical, I might not understand otherwise.”
Internal and external support systems affecting SDM	Dual nature of family support	“My family pays close attention to me and reminds me to proactively share my concerns with the doctor.” “For many patients after they fall ill, some family members have a positive attitude … When we invite them to participate together, they are usually very proactive and enthusiastic.” “Families of patients with mental disorders in China believe they have an obligation to care for the patient … But sometimes the patient’s parents cannot understand the medical staff’s actions … making it difficult to provide objective information, and the patient finds it hard to truly participate in decision-making.”
	Sociocultural and individual beliefs	“If people found out about my condition, it would be deeply shameful.” “Compared to developed countries like those in Europe and America, the level of discrimination against mental patients in China is higher. Such patients are often labeled with derogatory terms like “crazy” or “idiot” by outsiders, resulting in a greater sense of inferiority compared to other diseases.” “I also worry that others will think mental patients “lack insight,” so I simply remain silent … Could you tell the doctors that sometimes we nod in agreement on the surface, but it’s actually because of stigma that we don’t dare to contradict?”

### 3.1 Perceptions of SDM and role positioning

#### 3.1.1 Value recognition and role multiplicity

Both nurses and patients generally recognized the positive significance of patient involvement in SDM. Nurses viewed it as an important approach to enhance treatment adherence and patient self-identity. As N3 stated, “Involving patients in decision-making can improve their self-identity and promote more in-depth communication.”

During the SDM process, nursing staff demonstrated multiple key roles. They not only acted as facilitators of decision-making but also served as coordinators between healthcare providers and patients, as well as advocates for patients’ rights. N2 emphasized, “ It is essential to comprehensively evaluate treatment options and properly guide patients toward suitable choices,” while N4 described their role as “building a communication bridge among patients, families, and doctors.” N11 further highlighted, “When patients’ rights are compromised, nurses must fully protect them.”​Additionally, the unique context of psychiatric care further assigns nurses the roles of crisis intervenors and long-term rehabilitation planners. As N8 explained, “When patients experience emotional fluctuations, rapid response and reassurance are necessary,” and N2 added, “We assist patients in jointly developing long-term care and rehabilitation plans.”

#### 3.1.2 Patients’ perceived capacity and decision-making experience

From the patient’s perspective, their engagement in decision-making was closely linked to their clinical status. When psychiatric symptoms were effectively controlled, patients demonstrated greater willingness and capacity to participate. As P6 described, “Recently, my mind feels much clearer, and I am more willing to express my thoughts.” Some patients adopted an open and proactive approach to communication, with P5 stating, “I hope to discuss my condition with the doctor together.”

However, when experiencing hallucinations, delusions, or thought disorganization, participation in decision-making became challenging. P3 acknowledged, “Sometimes my mind is chaotic, and the environment feels unsafe.” Furthermore, patients’ health beliefs directly influenced their level of engagement. Those with restored insight and growing confidence in treatment were more actively involved, as reflected by P11’s comment: “As long as I cooperate well with the treatment, I believe it will help.” In contrast, patients who lacked accurate awareness of their illness tended to reject participation, exemplified by P3’s remark: “I really don’t have an illness and don’t need any treatment.”

### 3.2 Attitudes and trust relationships influencing SDM implementation

#### 3.2.1 Diversity in nursing staff attitudes

Nursing staff attitudes toward SDM participation for patients with severe mental disorders fell into three categories: proactive supporters (n=7), who considered it a patient right and clinical responsibility—as N9 asserted, “Implementing SDM is a patient’s fundamental right!”; cautiously neutral practitioners (n=3), who acknowledged its value but expressed reservations about full implementation due to concerns over insufficient systemic support, exemplified by N7’s comment, “We must recognize that a mature SDM support system has not yet been established”; and explicitly opposed individuals (n=2), who primarily questioned patients’ decision-making capacity and perceived potential risks, summarized by N8’s statement, “Patients’ judgment differs significantly from the norm, making it difficult for them to reach sound decisions.”

#### 3.2.2 Central role of trust relationships

A trusting relationship among clinicians, nurses, and patients serves as the cornerstone for successful SDM implementation. When healthcare providers demonstrated a friendly and respectful attitude, patient engagement was significantly enhanced. As P11 shared, “The doctor was very patient and adjusted my medication promptly—I really valued that.” P9 also noted, “The doctors and nurses are highly professional; I feel reassured following their guidance.”

Conversely, when clinicians assumed full control due to perceived lack of decision-making capacity in patients, or when patients felt ignored, a crisis of trust emerged, hindering SDM participation. P10 expressed, “No one explained what medications I was taking, and they seemed impatient when I asked.” P8 added, “When I reported physical discomfort, the doctors and nurses didn’t believe me.” This power imbalance suppressed patients’ willingness to engage, as captured by P6’s remark: “I was afraid of bothering them, so I simply stopped speaking up.”

### 3.3 Practical challenges in SDM implementation

#### 3.3.1 Clinical and ethical contexts

Shared decision-making in psychiatric settings inherently involves complex risk-benefit assessments. As N10 highlighted, “Medication may lead to either improvement or deterioration of symptoms. When patients’ judgment is impaired, SDM becomes particularly challenging.” Furthermore, discrepancies between patients’ and family members’ preferences further complicate the decision-making process. N3 illustrated this dilemma: “The patient refused electroconvulsive therapy, while the family strongly advocated for it. Balancing these conflicting perspectives proved challenging.”

#### 3.3.2 Barriers to information communication

Effective information transfer is a prerequisite for shared decision-making, yet multiple barriers were encountered in practice. Patient P1 reported, “The doctor said it might cause ‘tardive dyskinesia,’ but I didn’t understand what that meant,” reflecting how professional terminology hindered comprehension. P9 highlighted issues of information overload and rushed delivery, noting, “The nurse explained too much too quickly—I could never remember everything.” In contrast, using visual aids such as illustrations and videos significantly improved understanding. As P6 affirmed, “After watching a short video on choking prevention, I remembered the instructions almost immediately.”

### 3.4 Internal and external support systems affecting SDM

#### 3.4.1 Dual nature of family support

Families play a dual role in the decision-making process. Supportive family involvement can effectively facilitate SDM, as illustrated by P6: “My family pays close attention to me and reminds me to proactively share my concerns with the doctor,” while also providing critical information to support decision-making. However, paternalistic substitution and coercion can severely undermine patients’ autonomy. P10 expressed frustration: “My family can discuss treatment options directly with the doctor—I don’t need to be involved.” P8 also shared: “My father insisted on electroconvulsive therapy for me, even though it gives me terrible headaches each time.”

#### 3.4.2 Sociocultural and individual beliefs

The social stigma surrounding mental illness exerts significant psychological pressure on patients, hindering their active participation. As P1 expressed, “If people found out about my condition, it would be deeply shameful.” Such discrimination may lead to self-stigmatization and avoidance of engagement. In contrast, patients who view their condition rationally and maintain confidence in their treatment are more likely to exercise their decision-making rights proactively. P7 affirmed, “I don’t believe having a mental illness is shameful. Choosing my treatment is my right.”

Although presented separately, these four themes are not discrete but rather interconnected, each influencing and being influenced by the others in shaping SDM implementation in inpatient psychiatric settings. The synthesis of these themes reveals a dynamic process in which conceptual readiness, trust-based therapeutic alliances, communication effectiveness, and systemic support interact synergistically. At the core of this process lies a dual transformation: nurses transitioning from care executors to skilled facilitators, and patients transitioning from passive recipients to active participants. This transformation unfolds within trusting nurse-patient relationships, is challenged by practical barriers including fluctuating cognitive capacity and communication difficulties, and is moderated by contextual factors such as family involvement and societal stigma.

## 4 Discussion

The synthesis of our findings reveals that SDM implementation in schizophrenia inpatient care is fundamentally a dynamic, relational process shaped by the synergistic interaction of four interconnected domains. At its core, SDM requires a dual transformation: nurses must move beyond traditional care-executor roles to become skilled facilitators, rights advocates, and crisis intervenors, while patients must be dynamically assessed and progressively activated to transition from passive recipients to active participants. This dual transformation unfolds within a trusting nurse-patient relationship, which serves as the foundation for meaningful engagement; without trust, even well-intentioned efforts to involve patients may be met with resistance, silence, or passive compliance. However, this process is constantly challenged by practical barriers—including patients’ fluctuating cognitive capacity, clinicians’ divergent attitudes toward patient competence, and ineffective information communication—which together create significant obstacles to SDM. These challenges are further moderated by contextual support systems: families exert dual influences of facilitation and paternalistic substitution, while societal stigma discourages active participation. Successful SDM implementation thus depends on the alignment of these four domains: conceptual readiness and patient activation, sustained by trust-based relationships, navigated through practical challenges, and supported or hindered by family and social systems. When these elements align, SDM becomes not merely a clinical procedure but a transformative therapeutic process that enhances patient autonomy, strengthens therapeutic alliances, and promotes recovery-oriented outcomes.

### 4.1 Reconstructing core concepts: from role empowerment to patient activation

This study confirms that promoting the implementation of Shared Decision-Making (SDM) in psychiatric settings primarily requires a dual transformation in core concepts. On one hand, it involves expanding and empowering the role of nursing staff. The findings reveal that psychiatric nurses have transcended their traditional role as mere executors of care, evolving into multifaceted identities such as decision-making facilitators, rights advocates, and crisis intervenors. This aligns with research by Nagayama (17), who identified that psychiatric nurses engage in a dynamic process of “sensing responses to triggering stimuli” and creating opportunities for patients’ self-understanding and self-selection, while proactively taking preventative measures when necessary. Perkins et al. (18) further demonstrated that guided SDM training can effectively increase clinicians’ use of SDM in mental health settings. Through systematic training, such as SDM workshops and scenario simulations, supported by decision aids and structured institutional protocols, nurses can comprehensively enhance their practical SDM skills and confidence, thereby translating conceptual approval into effective clinical practice (18).

On the other hand, it necessitates the “activation” of patients and the establishment of their central role in care. Research indicates (19) that the decision-making capacity of patients with severe mental illness is not static but fluctuates dynamically with symptom changes and the recovery of insight, a finding consistent with the results of this study. This requires healthcare providers to move beyond viewing patients as a homogeneous group and instead adopt a model of dynamic assessment and collaborative empowerment. By evaluating patients’ willingness and ability to participate in decision-making at different stages, providers can guide them progressively from being “passive recipients” to becoming “active participants,” thereby helping restore their sense of self-worth. This “activation” process emphasizes the discovery and enhancement of individuals’ intrinsic capabilities, helping patients build confidence and improve self-management skills through information provision, emotional support, and skill development (20).

### 4.2 Building trust relationships: overcoming power imbalances and cognitive biases

This study found that some healthcare providers, influenced by a tendency toward “self-oriented” cognition, harbor doubts about patients’ decision-making capacity. This can manifest as authoritative dominance during communication, directly leading to patients’ sense of alienation and a crisis of trust, making them feel their opinions are disrespected (21). Qualitative research shows (22) that “being seen and understood” is foundational to the patient experience of SDM, and a trusting relationship is its core (23). Existing studies confirm (24) that for patients with severe mental disorders, factors such as diagnosis, symptom severity, or illness duration do not significantly hinder their participation in SDM. Notably, a negative correlation has even been observed between patients’ level of insight and their preference for autonomous decision-making (25). In clinical practice, it is crucial for healthcare providers to fully recognize the importance of maintaining open communication with patients and respecting their personal dignity. Efforts should be made to establish a therapeutic alliance wherein doctors, nurses, and patients are partners with shared goals, achieving power-sharing through candid dialogue and equal negotiation (26). Such a robust trusting relationship not only enhances patient engagement but also possesses intrinsic therapeutic value.

### 4.3 Optimizing communication processes: building an “adapted and accessible” information support system

Effective SDM is built on the foundation of bidirectional and equitable information exchange. However, this study highlights specific challenges within psychiatric settings. Cognitive impairments associated with the illness itself, coupled with healthcare providers’ frequent reliance on professional jargon for rapid, one-way information transmission due to time constraints, result in patient misunderstanding and low information retention, ultimately hindering effective participation in decision-making (7, 8). To address this, there is an urgent need to establish “diversified and adapted” information communication channels. A key future direction is the active development and introduction of patient decision aids specifically designed for psychiatry. Such tools can translate complex medical information into visual formats like charts and videos, lowering comprehension barriers, assisting patients in integrating their personal preferences, and embodying the relational, communicative, and supportive dimensions of SDM (27, 28).

### 4.4 Enhancing support systems: integrating family and social functions

This study reveals that disagreements occasionally arise between patients and their families. In China, professionals often tend to seek consent from family members rather than the patients themselves. Furthermore, it is sometimes perceived that patients being overly informed about treatment details may be detrimental to their recovery (29). Therefore, establishing a tripartite SDM model involving mental health professionals, patients, and patients’ families—a family-centered decision-making model (29–31)—is recommended to reduce or avoid conflicts in shared decision-making. Research in cross-cultural contexts suggests that for groups valuing interdependence and family collective interests (e.g., within Asian cultures), employing Family-Centered Decision-Making (FCDM) as a complement to SDM may represent a more culturally responsive collaborative approach (32).

Additionally, this study indicates that individuals with mental illness may face various negative consequences due to societal stigma, which hinders their participation in SDM. This finding is consistent with research by Nxumalo & Ngubane (33). Currently, China is further advancing the development of mental health services through annual policy initiatives (34). For example, some psychiatric institutions have adopted ‘Internet+’ models to promote whole-course management of patients with severe mental illness, extending care from hospitals to families, communities, and society. This provides a robust support system for facilitating patient participation in SDM.

From an implementation science perspective, the multilevel determinants identified in this study can inform targeted strategies for sustainable SDM implementation. At the provider level, systematic training programs are needed to address nurses’ divergent attitudes and enhance facilitation skills. At the patient level, flexible, stage-based activation strategies should respond to patients’ fluctuating capacity to participate. At the communication level, adapted decision aids (e.g., visual tools, simplified language) should be integrated into routine care. At the family level, structured family involvement protocols can harness family support while safeguarding patient autonomy. At the organizational level, institutional policies must mandate SDM training, allocate resources for decision aids, and establish accountability mechanisms. Importantly, these levels interact synergistically, suggesting that implementation efforts should adopt multi-component, systems-oriented approaches rather than targeting any single determinant in isolation.

### 4.5 Limitations

This study has several limitations. First, the single-site design and relatively small sample from one tertiary psychiatric hospital in China may limit the transferability of findings to other settings or populations. Although data saturation was achieved, participants were predominantly nurses with Bachelor’s degrees and patients with paranoid schizophrenia, potentially underrepresenting diverse perspectives across different clinical contexts, cultural backgrounds, or stages of illness. Second, the study focused exclusively on nurses and patients without including perspectives of psychiatrists, family caregivers, or other mental health professionals who significantly influence decision-making processes in psychiatric settings. Given the complex family dynamics and multidisciplinary nature of schizophrenia care identified in our findings, the absence of these stakeholders represents an important gap in understanding the full ecology of SDM implementation. Third, while Colaizzi’s seven-step phenomenological framework provided a systematic and transparent approach to analysis, its structured nature may have limited the interpretive depth achievable through more hermeneutic phenomenological approaches. Nonetheless, we consider Colaizzi’s method appropriate for this study given its clarity and systematic procedures, which facilitated rigorous analysis and transparent reporting. Fourth, the analysis of nurse interview data involved the interviewer as a co-analyst. While this approach was justified by the interviewer’s essential contextual expertise and safeguarded by independent critical review from a second researcher, we acknowledge that this may have introduced potential biases in data interpretation. Patient data, in contrast, were analyzed independently by coders not involved in data collection. All coding decisions were resolved through team consensus to maximize trustworthiness.

## 5 Conclusion

This study reveals that implementing shared decision-making for patients with schizophrenia is a complex process influenced by multilevel determinants spanning provider, patient, communication, family, and organizational domains. From an implementation science perspective, sustainable SDM integration requires coordinated strategies across all these levels: systematic provider training, flexible patient activation approaches, adapted decision aids, structured family involvement protocols, and institutional policies that mandate training and resource allocation. Building therapeutic alliances based on trust and integrating family support are essential to overcoming barriers. Future implementation efforts should adopt multi-component, systems-oriented approaches to assess local contexts and select appropriate strategies. Such efforts should also focus on culturally adapted interventions that address the unique challenges of psychiatric settings in China. This research informs health policy by highlighting the need for SDM training mandates, resource allocation for patient decision aids, and accountability mechanisms. For clinical nursing management, our study underscores the importance of institutional protocols that foster trust-based nurse-patient relationships, dynamic patient capacity assessment, and interdisciplinary collaboration—ultimately enhancing treatment adherence, patient autonomy, and recovery outcomes.

## Data Availability

The original contributions presented in the study are included in the article/**Supplementary Material**. Further inquiries can be directed to the corresponding authors.
