# Supplementary material for: Implementing shared decision-making for hospitalized patients with schizophrenia: a qualitative study of nurse and patient perspectives
Source: Front Psychiatry. 2026 Aug 6;17:1873569. doi: 10.3389/fpsyt.2026.1873569 (PMC13490086; doi:10.3389/fpsyt.2026.1873569)
Supplement: Supplementary file 1 [file SupplementaryFile1.doc]

**Interview Guides**

**1. Nurse Interview Guide:**

- How do you view the involvement of patients with mental disorders in clinical decision-making, based on your work experience?
- In your practice, what have you observed regarding the participation of patients with mental disorders in shared clinical decision-making?
- What factors do you think influence patients' participation in shared clinical decision-making?
- Based on your experience and observations, what suggestions do you have to facilitate more effective participation of patients with mental disorders in shared clinical decision-making?
- Is there anything else you would like to add or share regarding this interview?

**2. Patient Interview Guide:**

- Are you familiar with shared decision-making (SDM)? What do you think it generally involves?
- Could you describe your experience and feelings regarding participation in treatment and nursing care decisions (SDM)?
- What do you perceive as barriers and facilitators in the decision-making process?
- What suggestions or opinions do you have regarding participation in clinical SDM?
- Is there anything else you would like to add regarding this interview?
